# Genome Sequence of *Enterococcus faecalis* Strain CG_E

**DOI:** 10.1128/genomeA.01488-16

**Published:** 2017-01-12

**Authors:** Christina Gabris, Anja Poehlein, Frank R. Bengelsdorf, Rolf Daniel, Peter Dürre

**Affiliations:** a Institut für Mikrobiologie und Biotechnologie, Universität Ulm, Ulm, Germany; b Genomic and Applied Microbiology & Göttingen Genomics Laboratory, Georg-August University Göttingen, Göttingen, Germany

## Abstract

*Enterococcus faecalis* CG_E is a Gram-positive, lactic acid-producing coccus. The draft genome of *E. faecalis* strain CG_E comprises 2,969,881 bp and exhibits a G+C content of 37.34%. The genome encodes 2,848 predicted protein-encoding and 97 RNA genes.

## GENOME ANNOUNCEMENT

*Enterococcus faecalis* was isolated from peritoneal fluid in 1906 as a *Streptococcus* species (1), a designation which was revised in 1989 by Coykendall (2). This strain is known as a commensal bacterium that inhabits the gastrointestinal tract of humans and other mammals (3). Other studies also showed that 16S rRNA gene sequences of *Enterococcus* are also present in low abundances in biogas reactors (4).

*E. faecalis* strain CG_E was isolated from biogas reactor content and initially designated *E. faecalis* CG01 (5). Maize silage and cattle as well as poultry dry manure were the main substrates supplied to the reactor, which is located in Troisdorf, Germany (6).

Genomic DNA was isolated from *E. faecalis* CG_E cells cultured in RCM liquid medium (Oxoid Ltd., Basingstoke, Hampshire, United Kingdom) at 37°C under an N_2_ and CO_2_ (80% + 20%) atmosphere. For genome sequencing, we used an approach that combined both the Titanium chemistry of the 454 GS-FLX pyrosequencing system (Roche Life Sciences) and the Genome Analyzer IIx (2- × 112-bp paired-end sequencing; Illumina). Shotgun sequencing libraries were generated from the extracted genomic DNA according to the instructions of the manufacturers (Illumina, San Diego, CA, USA; Roche Life Science). Sequencing resulted in 10,316,210 paired-end Illumina reads (112 bp) that were trimmed using Trimmomatic 0.32 (7) and 76,327 454 pyrosequencing reads. For the hybrid *de novo* assembly performed with the Mira v 3.4 (8), four million quality-filtered Illumina reads and all 454 reads were used. This resulted in a draft genome consisting of 79 contigs with an average coverage of 152-fold. The genome of *E. faecalis* CG_E consists presumably of a single chromosome (2,969,881 bp) with a G+C content of 37.34%. For automatic gene prediction, the software tool Prodigal (9) was used. Genes coding for rRNA and tRNA were identified using RNAmmer (10) and tRNAscan (11), respectively. The Integrated Microbial Genomes–Expert Review (IMG-ER) system (12) was applied for automatic annotation followed by manual curation using the Swiss-Prot, TrEMBL, and InterPro databases (13).

The draft genome encodes three rRNAs and 47 tRNAs. The genome harbors 2,101 predicted protein-encoding genes with deduced functions and 747 (26.23%) coding for hypothetical proteins.

*In silico* pairwise average nucleotide identity (ANI) (14) using version 4.540 deposited on an IMG/ER system (12) was used to analyze the genetic identity of the genome sequence of *E. faecalis* strain CG_E with *E. faecalis* ATCC 19433^T^ (ASDA00000000), the type strain of this species. This analysis yielded 99.04% sequence similarity of both genome sequences, which clearly showed that the isolate CG_E belongs to the species *Enterococcus faecalis*. Genome inspection also revealed that a gene encoding a fructose-1,6-bisphosphate aldolase, the key enzyme for homofermentative lactic acid fermentation, is present. We also identified several phosphotransferase systems specific for several sugars such as fructose, glucose, maltose, sucrose, trehalose, mannose, and cellobiose and also for mannitol, sorbitol, and galactitol as well as for *N*-acetyl-d-glucosamine and *N*-acetyl-galactosamine. The genome harbors genes encoding a mannose-6-phosphate isomerase, an l-rhamnose-isomerase, and an l-rhamnose mutarotase as well as a mannitol-1-phosphate-5-dehydrogenase.

### Accession number(s).

This whole-genome shotgun project has been deposited at DDBJ/ENA/GenBank under the accession no. MIQF00000000. The version described in this paper is version MIQF01000000.

## References

[1] Andrewes FW, Horder TJ 1906 A study of the streptococci pathogenic for man. Lancet 168:708–713. doi:10.1016/S0140-6736(01)31538-6.

[2] Coykendall AL 1989 Rejection of the type strain of *Streptococcus mitis* (Andrewes and Horder 1906): request for an opinion. Int J Syst Bacteriol 39:207–209. doi:10.1099/00207713-39-2-207.

[3] Ryan KJ, Ray CG 2004, Sherris medical microbiology, 4th ed. McGraw-Hill, New York, NY.

[4] Li A, Chu Y, Wang X, Ren L, Yu J, Liu X, Yan J, Zhang L, Wu S, Li S 2013 A pyrosequencing-based metagenomic study of methane-producing microbial community in solid-state biogas reactor. Biotechnol Biofuels 6:3. doi:10.1186/1754-6834-6-3. 23320936 PMC3618299

[5] Gabris C 2015 Analysis of hydrolysis, acidogenesis, and methane oxidation in biogas reactors. PhD Thesis University of Ulm.

[6] Gabris C, Bengelsdorf FR, Dürre P 2015 Analysis of the key enzymes of butyric and acetic acid fermentation in biogas reactors. Microb Biotechnol 8:865–873. doi:10.1111/1751-7915.12299. 26086956 PMC4554474

[7] Bolger AM, Lohse M, Usadel B 2014 Trimmomatic: a flexible trimmer for Illumina sequence data. Bioinformatics 30:2114–2120. doi:10.1093/bioinformatics/btu170. 24695404 PMC4103590

[8] Chevreux B, Wetter T, Suhai S 1999 Genome sequence assembly using trace signals and additional sequence information, p. 45–56. *In* Wingender E (ed) Computer science and biology: proceedings of the German Conference on Bioinformatics (GCB) 1999, Hannover, Germany. GBF-Braunschweig, Department of Bioinformatics, Braunschweig, Germany.

[9] Hyatt D, Chen GL, Locascio PF, Land ML, Larimer FW, Hauser LJ 2010 Prodigal: prokaryotic gene recognition and translation initiation site identification. BMC Bioinformatics 11:119. doi:10.1186/1471-2105-11-119. 20211023 PMC2848648

[10] Lagesen K, Hallin P, Rødland EA, Staerfeldt H-H, Rognes T, Ussery DW 2007 RNAmmer: consistent and rapid annotation of ribosomal RNA genes. Nucleic Acids Res 35:3100–3108. doi:10.1093/nar/gkm160. 17452365 PMC1888812

[11] Lowe TM, Eddy SR 1997 TRNAscan-SE: a program for improved detection of transfer RNA genes in genomic sequence. Nucleic Acids Res 25:955–964. doi:10.1093/nar/25.5.0955. 9023104 PMC146525

[12] Markowitz VM, Chen IM, Palaniappan K, Chu K, Szeto E, Pillay M, Ratner A, Huang J, Woyke T, Huntemann M, Anderson I, Billis K, Varghese N, Mavromatis K, Pati A, Ivanova NN, Kyrpides NC 2014 IMG4 version of the integrated microbial genomes comparative analysis system. Nucleic Acids Res 42:560–567. doi:10.1093/nar/gkt963. PMC3965111 24165883

[13] Zdobnov EM, Apweiler R 2001 InterProScan--an integration platform for the signature-recognition methods in InterPro. Bioinformatics 17:847–848. doi:10.1093/bioinformatics/17.9.847. 11590104

[14] Kim M, Oh HS, Park SC, Chun J 2014 Towards a taxonomic coherence between average nucleotide identity and 16S rRNA gene sequence similarity for species demarcation of prokaryotes. Int J Syst Evol Microbiol 64:346–351. doi:10.1099/ijs.0.059774-0. 24505072

